# Prise en charge chirurgicale des varices des membres inférieurs: étude observationnelle rétrospective au Centre Hospitalier Régional Hassan II d'Agadir, Maroc

**DOI:** 10.11604/pamj.2025.52.150.41608

**Published:** 2025-12-09

**Authors:** Badr El Kassimi, Abdelkarim Kharroubi

**Affiliations:** 1 Service de Chirurgie Vasculaire et Endovasculaire, Centre Hospitalier Universitaire Souss Massa, Faculté de Médecine et de Pharmacie d'Agadir, Université Ibn Zohr Agadir, Maroc

**Keywords:** Varices, membres inférieurs, crossectomie, stripping, phlébectomie, Varicose veins, lower limbs, crossectomy, stripping, phlebectomy

## Abstract

Les varices sont définies comme des dilatations veineuses qui deviennent tortueuses. C'est une affection fréquente dans les pays industrialisés; elle serait présente chez 30 à 40% de la population générale. Les varices constituent un motif fréquent de consultation dans notre pratique. Il s'agit d'une étude rétrospective de série de cas, réalisée au Centre hospitalier régional Hassan II d'Agadir, entre avril 2021 et septembre 2022. Tous les patients ayant été opérés durant cette période pour des varices ont été inclus. Les données des patients ont été extraites à partir des dossiers des malades. Les variables analysées ont été: l'âge, le sexe, le motif de consultation, la classification des malades selon CEAP et l'intervention chirurgicale réalisée. Au total 15 patients ont été opérés, avec un âge moyen de 43,60±9,070 ans, des extrêmes de 28-62 ans, et un sex ratio égal à 1,14. Le principal motif de consultation était la phlébalgie associée à la lourdeur des membres inférieurs chez 5 (33,3%) malades, et 10 (66,7%) avaient consulté au stade C2. L'origine a été une varice essentielle chez tous les malades. Le geste le plus réalisé a été la crossectomie (ligature de la jonction saphéno-fémorale) associée à un stripping chez 8 (53,3%) patients. Les suites opératoires étaient favorables, avec une amélioration clinique sans aucune complication. La prise en charge des varices des membres inférieurs dans notre contexte est majoritairement chirurgicale, dont le geste le plus réalisé est la crossectomie associée ou non au stripping.

## Abstract (en)

Varicose veins are defined as venous dilatations that become tortuous. This is a common condition in industrialised countries, affecting 30 to 40% of the general population. Varicose veins are a common reason for consultation in our practice. We conducted a retrospective case series study at the Hassan II Regional Hospital of Agadir, between April 2021 and September 2022. All patients who underwent surgery for varicose veins during this period were included. Patient data were extracted from medical records. The variables analyzed were: age, sex, reason for consultation, patient classification according to CEAP, and the surgical procedure performed. A total of 15 patients underwent surgery, with an average age of 43.6060±9.070 years, ranging from 28 to 62 years, and a sex ratio of 1.14. The main reason for consultation was phlebalgia associated with heaviness of the lower limbs in 5 patients (33.3%), and 10 patients (66.7%) had consulted at stage C2. The origin was primarily varicose veins in all patients. The most common procedure performed was crossectomy (ligation of the saphenofemoral junction) associated with stripping in 8 patients (53.3%). The postoperative outcomes were favourable, with clinical improvement and no complications reported. In our setting, varicose veins of the lower extremities are managed primarily with surgical intervention, most commonly crossectomy, either alone or in combination with stripping.

## Introduction

Les varices sont définies comme des dilatations veineuses qui deviennent tortueuses. De symptomatologie variée, elles peuvent ne présenter aucune gêne pour le patient, comme peuvent être associées à des lourdeurs au niveau du membre inférieur atteint, un œdème, des démangeaisons de la peau, et parfois un ulcère variqueux au niveau des chevilles. C'est une affection fréquente dans les pays industrialisés; d'après les données épidémiologiques, elle serait présente chez 30 à 40% de la population générale [1]; cette prévalence augmenterait avec l'âge. Selon une étude américaine, sa prévalence serait de 10,4-23,0% et de 29,5-30,0%; respectivement chez les hommes et les femmes [2]. Les varices constituent un motif fréquent de consultation dans notre pratique et une part non négligeable des pathologies vasculaires observées. Cette étude visait à décrire la prise en charge chirurgicale des varices dans notre établissement hospitalier et à comparer les modalités thérapeutiques chirurgicales avec la littérature.

## Méthodes

**Conception et cadre de l'étude:** nous avons réalisé une étude observationnelle transversale rétrospective et descriptive au service de chirurgie vasculaire au Centre Hospitalier Régional Hassan II, Agadir, Maroc. Cela consistait à comparer les résultats des patients opérés pour varice.

**Population étudiée:** tous les patients ayant été opérés entre avril 2021 et septembre 2022 pour des varices des membres inférieurs ont été inclus.

**Collecte de données:** les données des patients ont été extraites à partir des dossiers des malades. Toutes les données collectées ont été traitées de manière confidentielle; les dossiers ont été numérotés sans aucune information nominative dans la fiche d'exploitation pour garantir l'anonymat des patients.

**Participants: *Critères d'inclusion:*** tous les patients opérés pour varice et ayant bénéficié d'un traitement chirurgical. ***Critères d'exclusion:*** les patients qui ont bénéficié d'un traitement médical seul. Au total, 15 patients ont été retenus.

### Définitions

***Clinique, Étiologie, Anatomie et Physiopathologie (CEAP):*** acronyme de la classification CEAP pour les insuffisances chroniques superficielles, un système standardisé internationalement qui utilise quatre catégories pour décrire les affections veineuses chroniques.

***Crossectomie:*** consiste à ligaturer la crosse de la veine saphène au niveau de sa jonction avec le réseau veineux profond.

***Phlébectomie:*** technique chirurgicale mini-invasive pour retirer les varices superficielles à l'aide de petites incisions cutanées et d'un crochet.

**Analyse statistique:** l'analyse des données a été faite par le logiciel JAMOVI 2.6.44 en utilisant la rubrique “analyse descriptive”. Les critères que nous étudions sont âge, le sexe, le motif de consultation, la classification des malades selon CEAP, et l'intervention chirurgicale réalisée.

**Considérations éthiques:** nous avons obtenu le consentement éclairé de tous les participants que nous avons opérés, conformément aux normes éthiques et réglementaires. Le comité d'éthique local a accordé l'approbation éthique.

## Résultats

**Caractéristiques générales et comorbidités de la population étudiée:** au total 15 patients ont été opérés, avec un âge moyen de 43,60±9,070 ans, des extrêmes 28-62 ans, et un sex ratio égal à 1,14. Huit (53,3%) patients sont des femmes et 7 (46,7%) des hommes. Neuf (60%) patients sont obèses et 12 (80%) patients présentaient une varice de la veine grande saphène alors que 3 (20%) patients présentaient une varice de la veine petite saphène.

**Figure 1:**
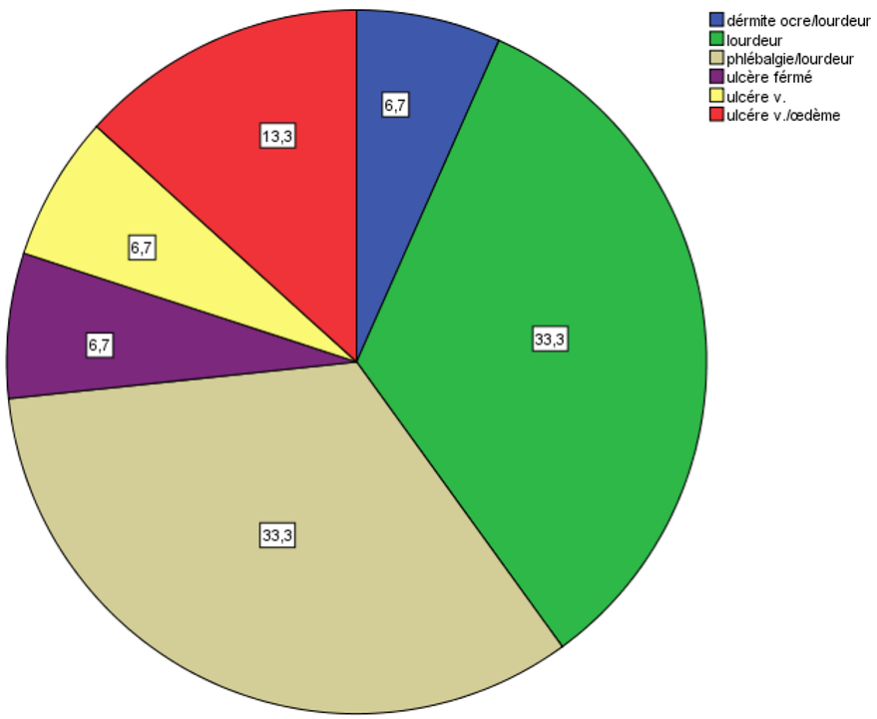
répartition des patients selon le motif de consultation

**Figure 2:**
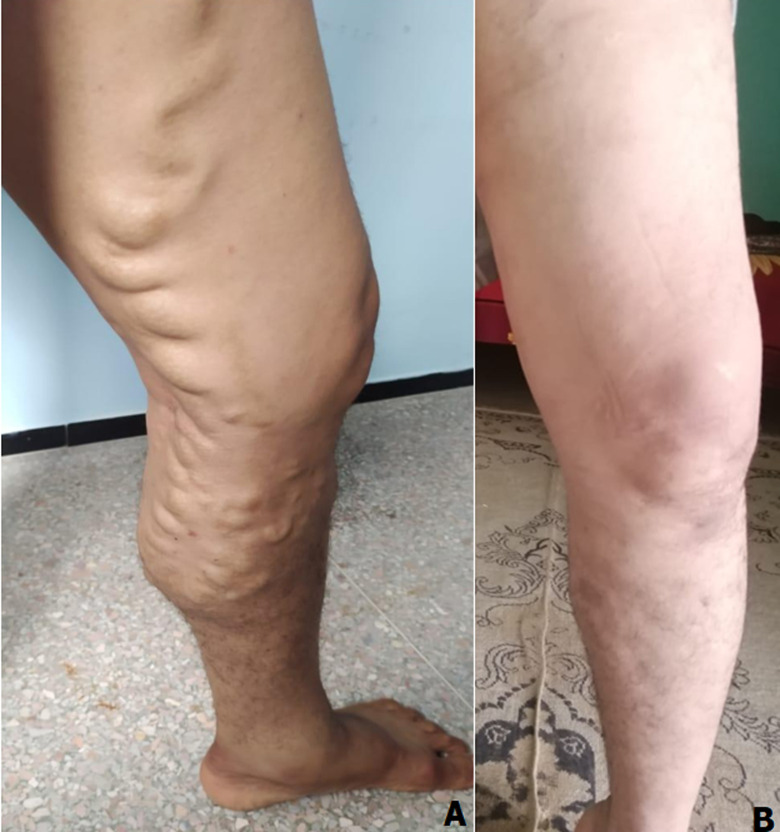
A) varice au stade C2 selon la classification CEAP; B) avec bonne évolution clinique après 7 mois de l'intervention chirurgicale

**Figure 3:**
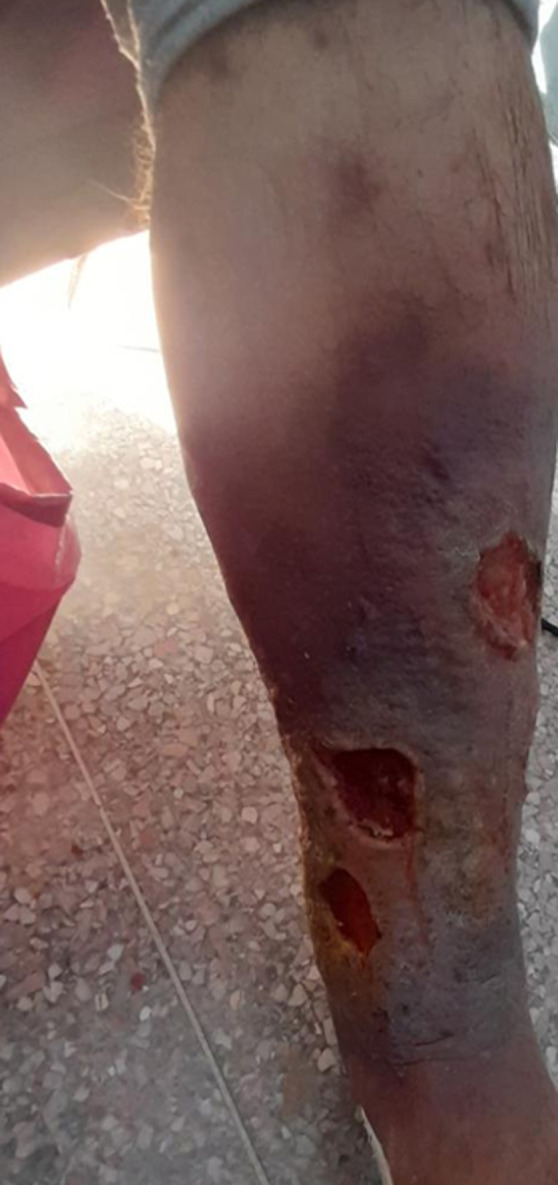
varice au stade C6 4a de la classification CEAP

**Figure 4:**
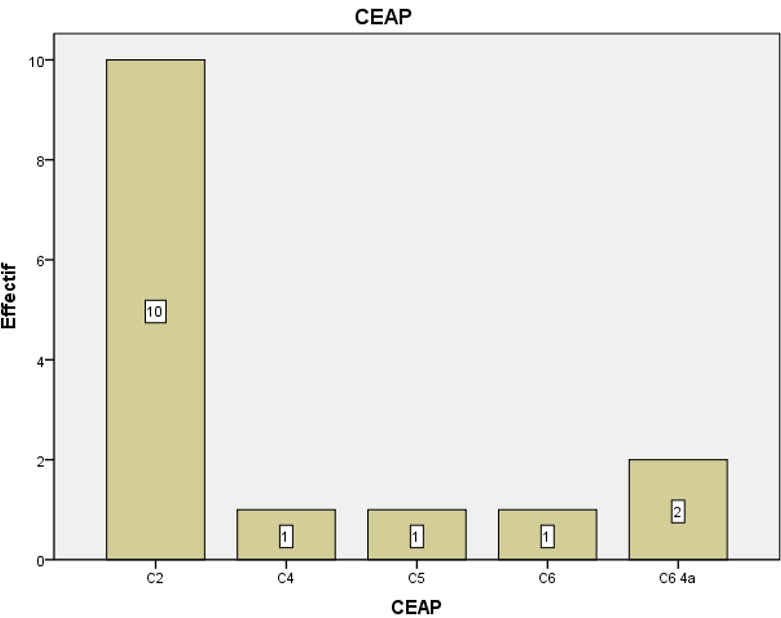
répartition des patients selon classification CEAP d'Hawaï modifié de 2004

**Données cliniques et paracliniques:** le principal motif de consultation était la phlébalgie associée à la lourdeur des membres inférieurs chez 5 (33,3%) malades, 5 (33,3%) patients avaient une lourdeur seule (Figure 1). Dix (66,7%) avaient consulté au stade C2 (Figure 2), 2 (13,3%) au stade C64a (Figure 3). Tous nos patients ont bénéficié d'une échographie Doppler veineux préopératoire des 2 réseaux (profond et superficiel) pour mesure de diamètre et évaluation de la continence (Figure 4). L'origine des varices a été essentielle chez tous les patients. Le réseau veineux profond est perméable et continu chez tous les patients.

**Figure 5:**
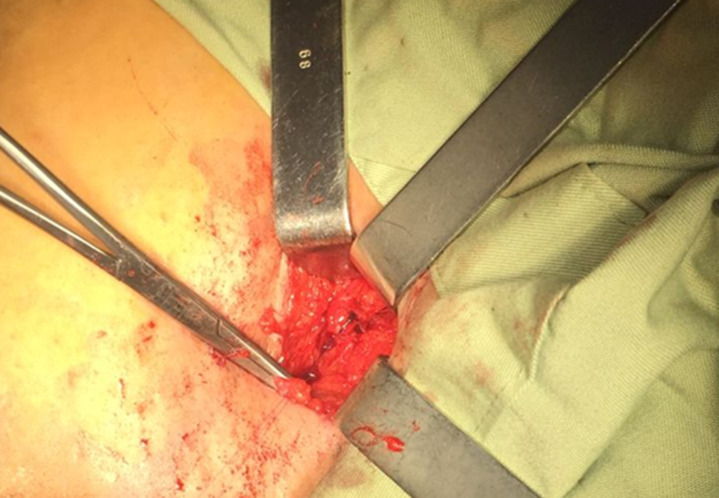
crossectomie de la veine grande saphène droite

**Figure 6:**
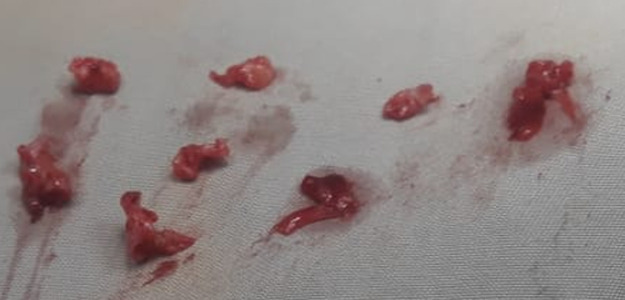
phlébectomie des paquets variqueux

**Figure 7:**
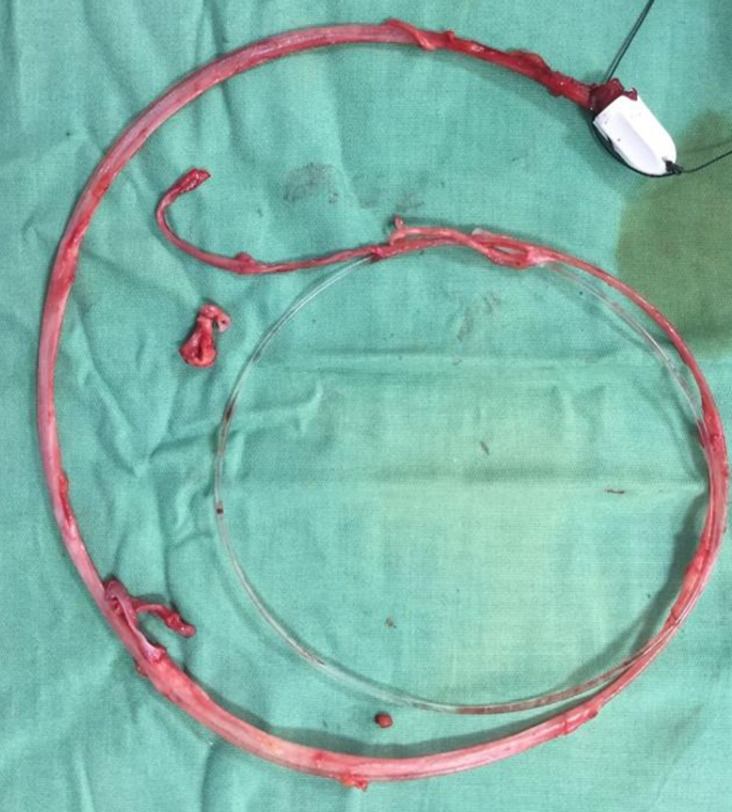
veine grande saphène strippé

**Prise en charge chirurgicale:** le geste le plus réalisé a été la crossectomie (ligature de la jonction saphéno-fémorale) (Figure 5) associée à un stripping chez 8 (53,3%) patients, suivie de crossectomie avec stripping (Figure 6) et phlébectomie chez 5 (33,3%) (Figure 7). La durée d'hospitalisation de nos malades est de 2 jours. L'antibiothérapie et l'héparinothérapie à base d'héparine de bas poids moléculaire (HBPM), à dose préventive, ont été systématiques pendant une semaine chez tous les patients. Une contention élastique systématique était réalisée à la fin de l'acte opératoire à l'aide d'une bande à contention. Le suivi à moyen terme en postopératoire des patients était de 1 à 1 mois et demi, avec une bonne évolution clinique (Figure 2) ainsi qu'aucun cas de complication ou de récidive n'a été signalé.

## Discussion

L'objectif de notre étude est de décrire la prise en charge chirurgicale des varices dans notre établissement hospitalier et de comparer les modalités thérapeutiques chirurgicales avec la littérature. Notre étude composée de 15 malades avec un âge moyen de 43.60±9,07 ans, des extrêmes 28-62 ans, et un sex ratio égal à 1,14 avec une légère prédominance féminine. L'atteinte de la veine grande saphène est la plus fréquente. Le principal motif de consultation était la phlébalgie associée à la lourdeur des membres inférieurs. La plupart de nos patients ont consulté au stade C2 de la classification CEAP. Tous nos patients ont bénéficié d'une échographie Doppler veineux préopératoire des 2 réseaux veineux.

Dans la majorité des études, il a été décrit une augmentation de la prévalence avec l'âge. Dans l'étude d'Evans *et al*. [3], la prévalence des varices tronculaires passe de 11,5% pour la tranche d'âge 18-24 ans à 55,7% pour celle de 55-64 ans. La plupart des travaux retrouvent une prévalence plus élevée des varices chez la femme que chez l'homme alors que certaines publications ne retrouvent pas cette différence. Dans un article publié par Widmer [4], il n'existe qu'une faible différence de pourcentage entre les sexes féminin et masculin (17%). Dans notre série, il existe une légère prédominance féminine, ce qui suggère l'intérêt de l'éducation et de la sensibilisation chez les femmes présentant des facteurs de risque des varices.

Dix de nos patients ont consulté au stade C2 (stade varice) de la classification CEAP, ce qui fait plus de 66% de toute la population d'étude. Cette consultation précoce, avant l'apparition des complications, peut être expliquée par la connaissance publique de cette pathologie de grande prévalence. Dans l'étude de De Brazzaville *et al*. plus d'un quart des patients ont consulté au stade C3 de la classification CEAP d'Hawaï modifiée, et 22,2% au stade C5-C6, avec des complications à type d'ulcère variqueux [5]. Ce recours précoce à la consultation spécialisée des formes débutantes des varices implique les bons résultats de la chirurgie des varices et limite l'apparition des troubles trophiques. La chirurgie était la base de notre conduite thérapeutique; à savoir la crossectomie associée au stripping et/ou à la phlébectomie. La crossectomie-stripping était le traitement de choix des varices jusqu'à ces dernières années avec l'arrivée de traitements endoveineux. Elle a encore de nombreuses indications [1].

La phlébectomie, réalisée avec stripping ou isolément, donne de bons résultats dans notre contexte pour le traitement des branches collatérales accessoires depuis Muller [6]. Elle est pratiquée grâce à de nombreuses multi-incisions à la lame de bistouri nº 11 et au crochet. Par biais technique dans notre structure, la sclérothérapie et le traitement endoveineux par radiofréquence et radiolaser n'étaient pas réalisables. Le choix de la méthode de traitement doit être en fonction de l'anatomie, de la physiopathologie et des comorbidités [7,8]. Certains auteurs présentent une stratégie thérapeutique dans l'ordre: les thérapies thermiques, suivies de la sclérothérapie, puis la chirurgie qu'en troisième position [9]; les recommandations internationales confirment le changement de hiérarchie dans le choix du traitement de l'incontinence variqueuse de la grande et de la petite veine saphène et la priorité donnée aux techniques endoveineuses d'ablation thermique, par rapport à la chirurgie conventionnelle [10].

L'obésité représente l'un des facteurs de risque les plus fréquents pour les troubles de la cicatrisation des plaies postopératoires, les nécroses cutanées et les infections [11]. Dans notre population, aucun malade avec une obésité n'a manifesté une infection de plaie en postopératoire du fait du changement de pansement correct et hygiénique. Les moyens de prévention de l'obésité par la promotion de l'activité physique et le suivi diététique permettent de diminuer la progression de la maladie variqueuse. Notre étude est la première sur la prise en charge chirurgicale des varices de notre région, ce qui apporte des données démographiques utiles. Elle fournit des résultats locaux qui permettent d'orienter la prévention de la maladie variqueuse. Les limites de notre travail apparaissent dans le petit nombre de patients inclus et le biais de sélection; seuls les patients opérés ont été inclus, excluant les patients ayant bénéficié du traitement médical.

## Conclusion

Notre étude descriptive rétrospective sur la prise en charge chirurgicale des varices des membres inférieurs dans le Centre hospitalier régional Hassan II nous a permis de dévoiler le profil épidémiologique et clinique local des patients opérés. Nos résultats montrent une prédominance féminine avec un sex ratio à 1,14 et une consultation au stade C2 de la classification CEAP. Le geste le plus réalisé est la crossectomie associée ou non au stripping. L'évolution était bonne sans récidive. Même si notre étude a des limites, elle fournit une base de référence locale pour l'amélioration de la prise en charge des varices.

### Etat des connaissances sur le sujet

- Les varices sont un motif fréquent de consultation, une maladie fréquente chronique qui traduit une insuffisance veineuse chronique superficielle;
- La classification CEAP permet de stadifier l'état des varices et de guider la prise en charge;
- Le stripping demeure la technique chirurgicale la plus utilisée dans certains centres avec des résultats meilleurs.

### Contribution de notre étude à la connaissance

- Notre étude fournit une base de référence locale sur le plan épidémiologique et clinique pour la prise en charge chirurgicale des varices;
- Notre étude met le point sur la place pertinente du stripping dans la prise en charge des varices à côté de traitements endoveineux;
- Ce travail ouvre la voie pour des études observationnelles prospectives avec un bon échantillon sur la prise en charge chirurgicale des varices.

## Available online at

https://www.panafrican-med-journal.com/content/article/52/150/full
